# Supplementary material for: Causal Relationship between Aging and Anorexia Nervosa: A White-Matter-Microstructure-Mediated Mendelian Randomization Analysis
Source: Biomedicines. 2024 Aug 16;12(8):1874. doi: 10.3390/biomedicines12081874 (PMC11351342; doi:10.3390/biomedicines12081874)
Supplement: Supplementary file 1 [file biomedicines-12-01874-s001.zip › Supplementary Figure/Figure S4/Singe SNP plot of MR analysis for TL to ubm-b-1866.pdf]

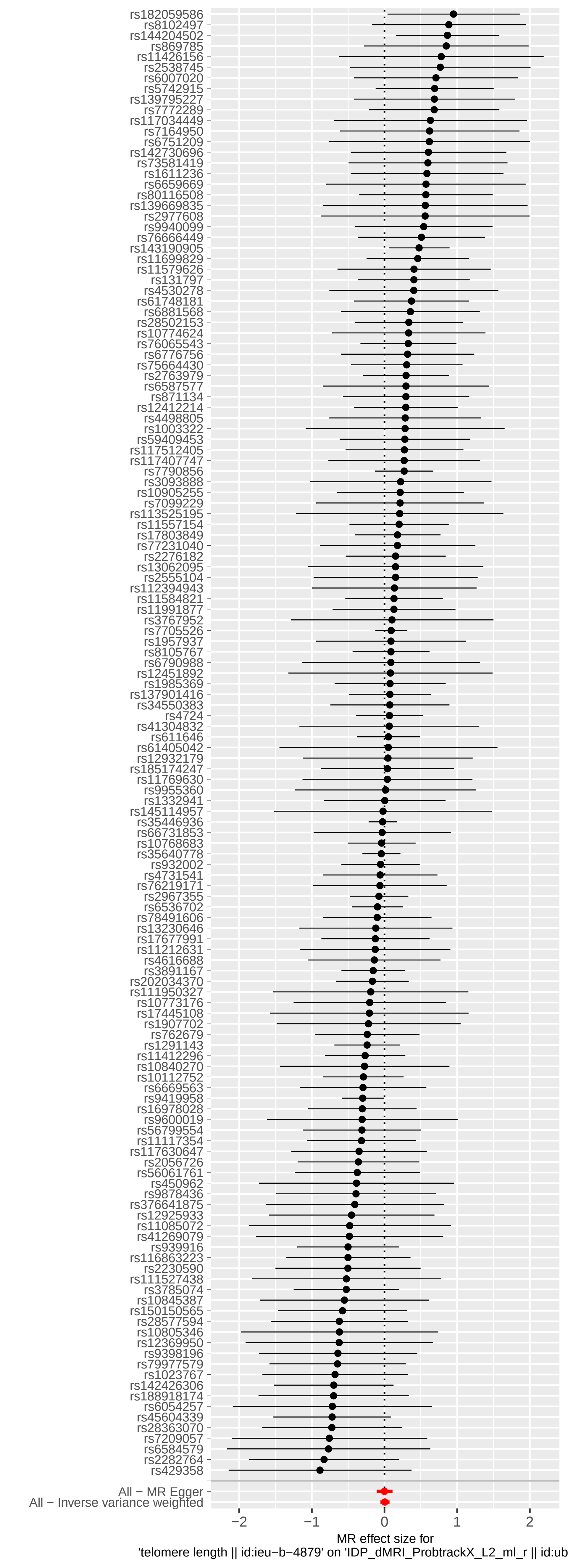
rs182059586
rs8102497
rs144204502
rs869785
rs11426156
rs2538745
rs6007020
rs5742915
rs139795227
rs7772289
rs117034449
rs7164950
rs6751209
rs142730696
rs73581419
rs1611236
rs6659669
rs80116508
rs139669835
rs2977608
rs9940099
rs76666449
rs143190905
rs11699829
rs11579626
rs131797
rs4530278
rs61748181
rs6881568
rs28502153
rs10774624
rs76065543
rs6776756
rs75664430
rs2763979
rs6587577
rs871134
rs12412214
rs4498805
rs1003322
rs59409453
rs117512405
rs117407747
rs7790856
rs3093888
rs10905255
rs7099229
rs113525195
rs11557154
rs17803849
rs77231040
rs2276182
rs13062095
rs2555104
rs112394943
rs11584821
rs11991877
rs3767952
rs7705526
rs1957937
rs8105767
rs6790988
rs12451892
rs1985369
rs137901416
rs34550383
rs4724
rs41304832
rs611646
rs61405042
rs12932179
rs185174247
rs11769630
rs9955360
rs1332941
rs145114957
rs35446936
rs66731853
rs10768683
rs35640778
rs932002
rs4731541
rs76219171
rs2967355
rs6536702
rs78491606
rs13230646
rs17677991
rs11212631
rs4616688
rs3891167
rs202034370
rs111950327
rs10773176
rs17445108
rs1907702
rs762679
rs1291143
rs11412296
rs10840270
rs10112752
rs6669563
rs9419958
rs16978028
rs9600019
rs56799554
rs11117354
rs117630647
rs2056726
rs56061761
rs450962
rs9878436
rs376641875
rs12925933
rs11085072
rs41269079
rs939916
rs116863223
rs2230590
rs111527438
rs3785074
rs10845387
rs150150565
rs28577594
rs10805346
rs12369950
rs9398196
rs79977579
rs1023767
rs142426306
rs188918174
rs6054257
rs45604339
rs28363070
rs7209057
rs6584579
rs2282764
rs429358
All − MR Egger
All − Inverse variance weighted
−2
−1
0
1
2
MR effect size for
'telomere length || id:ieu−b−4879' on 'IDP_dMRI_ProbtrackX_L2_ml_r || id:ub
